# Endoscopic ultrasound-free lumen apposing metal stent recanalization of a complete ileal J-pouch stenosis restoring intestinal continuity

**DOI:** 10.1055/a-2767-0722

**Published:** 2026-01-15

**Authors:** Pietro Graceffa, Alba Sparacino, Emanuele Bracciamà, Fabio Cartabellotta, Cosimo Callari, Antonino Granata

**Affiliations:** 1 213309 Interventional Endoscopic Unit, Buccheri La Ferla Hospital, Palermo, Italy; 2 213309 Department of Internal Medicine, Buccheri La Ferla Hospital, Palermo, Italy; 3 213309 Center of Excellence in Bariatric Surgery, Buccheri La Ferla Hospital, Palermo, Italy

Benign strictures after ileal pouch–anal anastomosis occur at the pouch inlet, mid-pouch, or anastomosis and may impair pouch function and quality of life. Endoscopic therapies – including balloon dilation and stricturotomy – are effective and organ-sparing, but recurrence and technically inaccessible strictures remain challenging 1 2 . Self-expanding metal stents have been explored in benign large-bowel obstruction with encouraging efficacy and acceptable adverse events in selected patients 3 . For “complete” anastomotic occlusions, combined antegrade–retrograde “rendezvous” techniques have been described; more recently, lumen-apposing metal stents (LAMSs) have enabled endoscopic re-anastomosis, typically with EUS guidance 4 5 .

We present an EUS-free recanalization of a complete J-pouch afferent-limb stenosis ( Video 1 ).

EUS-free, wire- and fluoroscopy-guided deployment of a 16 × 20-mm LAMS across a complete J-pouch afferent-limb stenosis, restoring pouch continuity.Video 1

A 45-year-old man with a history of ulcerative colitis underwent total colectomy with ileal J-pouch construction 13 years prior, complicated by an anastomotic fistula requiring ileostomy. The patient subsequently developed chronic pouchitis for which vedolizumab therapy was initiated.

**Fig. 1:**
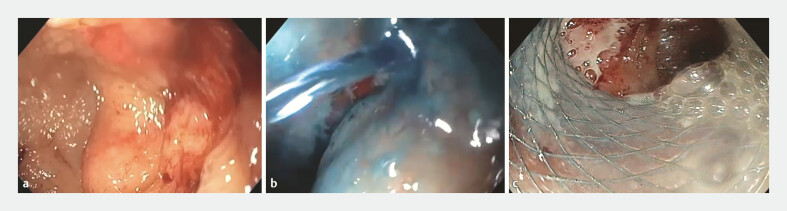
**a** An endoscopic view of complete pouch occlusion. **b** Fistulous tract identification. **c** Post-deployment traversal confirming restored patency.

A follow-up endoscopy demonstrated a complete stenosis of J-pouch with an afferent limb in the absence of an endoscopically visible communication ( Fig. 1 **a** ). An endoscopic exploration of the efferent limb through the stoma was therefore performed in an unsuccessful attempt to visualize the communication with the pouch. A diagnostic maneuver was conducted by injecting saline and methylene blue into the efferent limb through the stoma, resulting in dye visualization within the pouch, confirming the presence of a fistulous tract ( Fig. 1 **b** ). A guidewire was introduced from the pouch into the afferent limb under endoscopic guidance. In order to restore intestinal continuity, we first performed an endoscopic dilation of the fistulous tract of up to 15 mm and then a LAMS 16 × 20 mm (Niti-S HOT SPAXUS Stent) was safely deployed over a guidewire across the stenosis under fluoroscopic guidance. The exploration of the afferent limb through the LAMS confirmed the successful endoscopic reanastomosis ( Fig. 1 **c** ). The procedure was well tolerated and the patient subsequently successfully underwent complete recanalization with the removal of ileostomy.

The procedure was performed using a colonoscope with a 3.7 mm working channel. The SPAXUS stent is preloaded on an electrocautery-enhanced delivery system, featuring an electrocautery tip designed to penetrate the target organ tissue. The system has a 10 Fr delivery profile and a working length of 180 cm and is compatible with therapeutic endoscopes with a working channel diameter of 3.7 mm or larger.

This EUS-free, wire- and fluoroscopy-guided LAMS approach may offer a minimally invasive option to re-establish pouch continuity in select complex postoperative scenarios where conventional dilation or rendezvous is impractical. Careful selection and fluoroscopic control are essential.

Endoscopy_UCTN_Code_TTT_1AQ_2AF
